# HIV Stigma and Status Disclosure in Three Municipalities in Ghana

**DOI:** 10.5334/aogh.3120

**Published:** 2021-06-18

**Authors:** Awolu Adam, Adam Fusheini, Martin Amogre Ayanore, Norbert Amuna, Faith Agbozo, Nuworza Kugbey, Prince Kubi Appiah, Geoffrey Adenuga Asalu, Isaac Agbemafle, Bright Akpalu, Senam Klomegah, Abdulrazak Nayina, Doris Hadzi, Kingsley Afeti, Christopher Emmanuel Makam, Felix Mensah, Francis Bruno Zotor

**Affiliations:** 1 Department of Family and Community Health, School of Public Health, University of Health and Allied Sciences, Ho, Ghana; 2 Center for Health Literacy and Rural Health Promotion, P. O. Box GP1563, Accra, Ghana; 3 Department of Preventive and Social Medicine, Dunedin School of Medicine, P.O. Box 56, 9054, Dunedin, University of Otago, Dunedin, New Zealand; 4 Department of Health Policy Planning, and Management, School of Public Health, University of Health and Allied Sciences, Ho, Ghana; 5 Department of General Studies, School of Natural and Environmental Sciences, University of Environment and Sustainable Development, Somanya, Ghana; 6 Department of Epidemiology and Biostatistics, School of Public Health, University of Health and Allied Sciences, Ho, Ghana; 7 HIV Counseling and Voluntary Testing Center, Hohoe Municipal Hospital, Hohoe, Ghana

## Abstract

**Background:**

HIV-related stigma and HIV status disclosure are important elements in the continuous fight against HIV as these impact the prevention efforts and antiretroviral treatment adherence among people living with HIV/AIDS (PLWHA) in many communities.

**Objectives:**

The objectives of the study were to examine the prevalence and experience of various types of HIV-related stigma and HIV status disclosure among PLWHA in Volta region.

**Methods:**

A cross-sectional design was used to collect quantitative data from 301 PLWHA. Descriptive statistics were used to analyze and present data on socio-demographic variables. Correlation analysis was done to determine factors associated with HIV stigma and status disclosure while a Mann-Whitney U test was used to determine differences in internalized HIV stigma.

**Findings:**

The mean age of the participants was 44.82 (SD: 12.22), 224 (74.4%) were female, and 90% attained at least primary education. A Pearson *r* analysis revealed that ethnicity (r[299] = 0.170, p = 0.003), religious affiliation (r[299] = –0.205, p = 0.001) and social support (r[299] = 0.142, p = 0.014) significantly predicted disclosure of HIV status. Fear of family rejection (62%) and shame (56%) were reasons for non-disclosure of HIV status. A Mann-Whitney’s *U-test* revealed that females are more likely than males to internalize HIV stigma. Community-related HIV stigma in the form of gossip (56.1%), verbal harassment (30.9%), and physical harassment (8.6%) was reported.

**Conclusion:**

A high rate of HIV status disclosure was found with social support, ethnicity, and religious affiliation being the associated factors. Internalized HIV stigma is prevalent among PLWHA while community-related stigma impacts HIV status disclosure. Strengthening social support systems and implementing culturally appropriate educational interventions may help in reducing community-related HIV stigma.

## Introduction

More people are living with HIV/AIDS today than ever since the emergence of the disease in the early 1980s which is a result of scientific advances such as the development and accessibility to antiretroviral drugs and public health interventions that have contributed to extended life expectancy and improvements in the Quality of Life (QoL) of people living with HIV (PLHIV). Notwithstanding, HIV continues to be a global public health burden despite progress achieved in the fight against the epidemic. In 2018, there were 1.7 million new infections globally, 77,000 AIDS-related deaths, and 37.9 million PLHIV [1]. Sub-Saharan Africa remains the hardest-hit region with 61% of all new HIV infections globally [1]. Women are disproportionately affected accounting for 54% of all new infections in 2018 [1]. Although there appears to be a declining trend for new infections in Ghana, many people are already infected and living with the disease in the country. The national HIV prevalence rate in 2018 was 1.7(1.4–2.0) among people aged 15–49 with an estimated population of 330, 000 (280,000–390,000) people living with HIV [1]. However, there were regional and gender variations in the HIV prevalence in the country ranging from 2.66% in Brong Ahafo, the region with the highest prevalence to 0.39% in the North East region, the region with the lowest prevalence [2]. Regarding gender variation, an estimated 1.1%(0.8–1.3) males and 2.3(1.9–2.8) females were infected with the disease in Ghana in 2018 [1]. Overall, a total of 117,199 males and 217,514 females were infected in 2018 [2].

There have been declines in infection rates in Ghana which can be attributed to the introduction and implementation of policies, programs, and strategic goals at both national and international levels to combat the HIV epidemic. One such strategy has been to encourage HIV status disclosure as it is linked to reductions in infections through motivating HIV testing and change in sexual behavior [3]. For instance, in a mixed-method study of benefits of HIV status disclosure to children and adolescents in Ghana, HIV status disclosure helped in improving medication adherence and led to healthier and responsible behavior among infected adolescents [3]. Other studies have reported an increase in follow-up visits, use of condoms, ART adherence, and reduction in horizontal infection following status disclosure [4,5,6].

Rates of HIV status disclosure vary within and across countries, cultures, and socio-demographic variables from low to high disclosure rates. For instance, in Cape Coast, Ghana, researchers reported that 78.6% of 510 participants disclosed their HIV status to at least one individual. A 95% disclosure was reported among 995 women in Cape Town, South Africa [8], and 83.7% among 507 PLWHA in Kenya [9]. Low rates of HIV status disclosure (33.3%) were however reported in Lower Manya Krobo district [3]; in the Eastern Region of Ghana, and with both Accra and Kumasi (16.2%) among non-intervention group participants [10].

However, stigma and discrimination against PLWHA are widespread and constitute major drawbacks in fighting HIV/AIDS including testing and HIV status disclosure [11,12]. Researchers have reported HIV-related stigma, both internalized and experienced or expected, as a major challenge in HIV status disclosure in Ghana and elsewhere including guilt, shame, fear of abuse, and rejection among others [3,13,14,15]. Stigmatization and discrimination of PLWHA take many forms including rejection by their communities and family members, refusal to eat with, share bed, shake hands, blaming, and ostracism [12,16]. In the 2014 GDHS, for instance, participants’ level of stigma against PLWHA was assessed with questions regarding caring for HIV positive patients, buying fruits from HIV positive sellers, or allowing HIV positive teachers to teach children. Only 8% of women and 14% of men expressed positive attitudes overall to the stigma indicators [17]. HIV/AIDS stigma and discrimination have the potential to make PLWHA engage in risky sexual behavior to hide their HIV status from their sexual partners. Studies have shown evidence of some PLWHA engaging in unprotected sexual relations risking reinfection and the continuous spread of HIV [18,19,20].

HIV/AIDS stigma and discrimination and its impact on HIV status disclosure have not been extensively studied in the Volta region of Ghana and more so in Hohoe, Kpando, and Kadjebi municipalities. We examined HIV status disclosure and HIV-related stigma and its associated factors and the relationship between stigma and status disclosure. This was necessary as the Volta region has been impacted by the HIV/AIDS epidemic and recorded a 2.2% prevalence rate according to the 2014 national HIV and AIDS estimates [17].

## Methods and Materials

### Study design and population

A descriptive cross-sectional study was employed to collect data from a sample of 301 PLWHA HIV/AIDS clinics in Hohoe, Kpando, and Dodi Papase municipalities in the Volta region. The target population for this study was PLWHA registered and receiving ART treatment in the three mentioned municipalities. The inclusion criteria were that a participant had to be positively diagnosed with HIV and receiving ART at the HIV clinics. The choice of cross-sectional study design was based on the fact that many of the PLWHA receiving ART at these clinics are not permanently residing in those municipalities but travel from across the Volta and Oti regions and the country at large.

### Sample size determination and sampling

The sample size calculation was done based on the PLWHA population approximation of 2,500 at the study sites. This sample size was derived using the sample determination formula by Kothari [21,22]. The formula recommended a sample size of 384 people which we further increased by 10% to account for the non-response rate to 423. However, due to the non-response and removal of some questionnaires that lacked substantial information, we have included a 301(73.2%) questionnaire in the analysis.

Due to the nature of the study and the target population involved, convenience sampling was used to sample participants into the study. This is a non-probability sampling technique where targeted participants are reached out to at their convenient places and invited to participate in a research study [22,23]. This was considered the most appropriate for this study because of the fact it is impossible and unethical to go into the general population to recruit PLWHA into a study. The voluntary counseling and testing centers were the most convenient places for both PLWHA and researchers to meet. Data collection was done on scheduled clinic days which were Tuesdays and Thursdays for all three centers.

### Data collection and analysis

We used a semi-structured and pretested questionnaire written in English and translated for those who were not literate in English to collect data for the study from the participants. The data were analyzed and presented using a statistical package for social science (SPSS) version 20. Descriptive statistics were used to analyze data on HIV status disclosure, and HIV-related stigma. Bivariate correlation and Chi-square (*X^2^*) analysis were used to determine factors correlated with HIV status disclosure and internalized HIV-related stigma. A Mann-Whitney U-test was used to measure the differences in internalized HIV-related stigma between male and female participants. Many steps were taken to ensure that the study followed required ethical standards and procedures. Ethical review and approval were sought from Ghana Health Service Ethics Committee (GHSERC) with approval number GHSERC09/04/17 and it was only upon receipt of ethical clearance that we started data collection.

## Findings

The results presented here represent data from a sample of 301 PLWHA made up of 244(74.4%) females and 77(25.6%) males who were largely literate (90%) with at least primary education.

### HIV status disclosure and associated factors

One objective of this study was to examine HIV status disclosure and its associated factors. The results revealed that the majority (238[79%]) of the participants had their status disclosed to a family member. However, 63(21%) reported not disclosing their status to anybody to the best of their knowledge. For those participants whose HIV status was disclosed, 152(50.5%) personally informed the family member about his/her HIV positive status. A significant number, 122(40.5%) reported that their family member(s) got to know about their status at the hospital disclosed by hospital personnel. Nine percent (27) of the participants did not know how their family members became aware of their HIV status. We found that some participants disclosed their status to multiple people and so the percentages presented are relative. For example, 75(25%) disclosed their status to their spouses and parents, 76(25.2%) disclosed their status to a sibling, and 43 (14.3%) disclosed their status to their biological children.

#### Factors associated with HIV status disclosure

We performed bivariate correlation analysis to determine the variables that demonstrated a statistically significant association with HIV status disclosure. Only ethnicity, religious affiliation, and social support availability showed a statistically significant association with HIV status disclosure. A Pearson *r* analysis revealed that ethnicity (r[299] = 0.170, p = 0.003), religious affiliation (r[299] = –0.205, p = 0.001) and social support (r[299] = 0.142, p = 0.014) significantly predicted HIV status disclosure. The results are presented in ***Table 1***. In cross-tabulation analysis, Pearson *X^2^* revealed that Ewes (79.8%) were significantly more likely to disclose their HIV status compared to Guans (9.7%), Akans (5%), Hausa (0.4%), Kotokolis (0.8%), and foreign nationals (1.3%), p(0.04) < 0.05. Pearson *X^2^* revealed that compared to Muslims (5%) and ATR followers (1.3%), Christians (93.7%) were significantly more likely to disclose their HIV status (p(0.000) < 0.001). The results also showed that those with a medium social support availability index were more likely to disclose their HIV status than those with minimal or high social support availability index (p(0.05) = 0.05).

**Table 1:** Factors associated with HIV status disclosure.


		ETHNICITY OF PARTICIPANT	RELIGIOUS AFFILIATION	SOCIAL SUPPORT INDEX	HIV STATUS DISCLOSURE

Ethnicity of participant	Pearson Correlation	1	–0.312**	0.054	0.170**

	Sig. (2-tailed)		0.000	0.349	0.003

	N	301	301	301	301

Religious affiliation	Pearson Correlation	–0.312**	1	–0.054	–0.205**

	Sig. (2-tailed)	0.000		0.347	0.000

	N	301	301	301	301

Social support index	Pearson Correlation	0.054	–0.054	1	0.142*

	Sig. (2-tailed)	0.349	0.347		0.014

	N	301	301	301	301

HIV status disclosure	Pearson Correlation	0.170**	–0.205**	0.142*	1

	Sig. (2-tailed)	0.003	0.000	0.014	

	N	301	301	301	301


**. Correlation is significant at the 0.01 level (2-tailed). *. Correlation is significant at the 0.05 level (2-tailed).

### Reason for non-disclosure of HIV status

As indicated above, 63(21%) reported that they did not disclose their HIV status to anybody. These participants were then asked to give reasons for which they kept or wanted to keep their HIV status secret. In relative percentages, we found that fear of family rejection (62%), shame (56%), fear of community discrimination (43%), fear of news spreading (32%), and fear of being divorced (24%) were nondisclosure reasons. However, the majority (57%) maintained that they just wanted to keep their status secret to themselves.

### Examination of HIV stigma and discrimination

Another important objective of this study was to examine the HIV-related stigma and discrimination experienced by the participants in this study. To achieve this, different sets of questions were included that targeted different kinds of HIV-related stigma including internalized, family, community, and healthcare-related.

### Internalized HIV stigma

We used 10 Likert scale questions to assess the presence of internalized stigma among the participants and the results are presented in ***Table 2***. For this analysis, we combined the first two responses and the third and fourth responses respectively as yes and no to each of the questions. In each of the questions, we found that majority of the participants reported zero self-stigma or blame for their HIV positive status by choosing “not at all” as their response as demonstrated in the table. However, a minority but significant number demonstrated internalizing HIV stigma in each of the questions. For instance, 121(36%) felt that they brought shame to their families. Also, 138(41%) felt disgusted with themselves and 85(25.3%) felt they should avoid sharing utensils so as not to infect others with HIV.

**Table 2:** Internalized HIV stigma among study participants.


STIGMA STATEMENT	FREQUENCY	RELATIVE PERCENT

*How much do you feel that you should avoid holding a new infant because of your HIV?*		

Very much	48	15.9

Somehow much	23	7.6

Less	31	10.2

Not all	199	66.1

*How much do you feel that you should avoid feeding children because of your HIV?*		

Very much	36	11.9

Somehow much	26	8.6

Less	35	11.6

Not at all	204	67.7

*How much do you feel that you should avoid sharing dishes or glasses just in case someone might catch HIV from you?*		

Very much	42	13.9

Somehow much	27	8.9

Less much	36	11.9

Not all	196	65.1

*How much do you feel that you have brought shame to your family because you have HIV?*		

Very much	72	23.9

Somehow much	34	11.2

Less much	34	11.2

Not at all	161	53.4

*How much do you feel that you should avoid visiting people because of your HIV?*		

Very much	36	11.9

Somehow much	24	7.9

Less	29	9.6

Not at all	212	70.4

*How much do you feel that you have HIV because you have done wrong behaviors?*		

Very much	33	10.9

Somehow much	39	12.9

Less much	44	14.6

Not at all	185	61.4

*How much do you feel that you should avoid cooking for people because you have HIV?*		

very much	35	11.6

Somehow much	23	7.6

Less much	31	10.2

Not at all	212	70.4

*How much do you feel guilty about having HIV?*		

Very much	70	23.2

Somehow much	43	14.2

Less much	48	15.9

Not all	140	46.5

*How much do you feel disgusting because of your HIV?*		

Very much	73	24.2

Somehow much	47	15.6

Less much	43	14.2

Not all	138	45.8

*How much do you feel that you are paying for karma or sins because you have HIV?*		

Very much	20	6.6

Somehow much	35	11.6

Less much	52	17.2

Not all	194	64.4


To make more meaning, total internalized stigma was computed by assigning values to each response category and summing up the scores into four categories; zero, minimal, average, and high internalized HIV stigma. Zero internalized stigma was defined as choosing “not at all” for answers for the ten questions and scoring 40 on the Likert scale. A minimal internalized stigma was a score between 30 and 39 while average internalized stigma was scoring between 20 and 29. A high internalized HIV stigma was scoring between 10 and 19, which meant that the participant would have chosen very much or somehow much as his or her answer for the 10 questions. From the computations, we found that most (237[79%]) demonstrated internalized HIV stigma but with varying levels of high, average, and minimal stigma. For instance, only 29(9.6%) demonstrated a high level of internalized stigma, 80(26.6%) reported average self-internalized stigma. The majority (128[42.5%]) reported minimal internalized HIV stigma while 64(21.3%) reported zero self-internalized stigmas with their HIV positive status. The results are summarized in ***Table 3***.

**Table 3:** Internalized HIV stigma categorization.


	FREQUENCY	PERCENT

High internalized stigma	29	9.6

Average internalized stigma	80	26.6

Low internalized stigma	128	42.5

Zero internalized stigma	64	21.3

Total	301	100.0


In a bivariate correlation using eight independent variables, Pearson *r* analysis revealed that only the gender of the participants had a statistically significant correlation with internalized HIV stigma (r[299] = –0.127, p = 0.02). We then conducted a Mann-Whitney’s U-test to find out the exact relationship between gender and internalized stigma. Male participants scored a mean internalized rank of 166.56 higher than the mean score of females (145.65) which was statistically significant (U = 7426, male = 166.56, female = 145.65, p = 0.05, two-tailed). This confirmed a Pearson *X^2^* which showed that females (96.6%) compared to males (3.4%) were significantly more likely to internalize HIV stigma (p [0.02] < 0.05).

### Community and family-related HIV stigma

Apart from internalized stigma, the participants were asked if they experienced stigma and/or discrimination from their community members or institutions. The questions to assess community-related stigma and discrimination were both verbally and physically related. The results show that the participants experienced more verbal compared to physical stigma/discrimination. For instance, 169(56.1%) reported being gossiped about. Again, 93(30.9%) reported being verbally harassed by their community members while 26(8.6%) reported being physically harassed. Those who reported being physically assaulted were 14(4.7%). Six questions about regular family interactions were used to examine family-related stigma and the results show that only aggregated 4% of participants reported refusal of family members to eat with, sleep in the same room, and shared accommodation with them.

### Hospital and healthcare professional stigma against PLWHA

For PLWHA, healthcare institutions and professionals are critical to their survival and wellbeing and any form of stigma and/or discrimination from healthcare professionals can have serious negative health outcomes. The results show minimal discrimination or stigma experienced by participants in this study from healthcare professionals. For each of the questions, less than 5% of the participants reported experiencing stigma or discrimination from healthcare professionals with an aggregate of 3% prevalence.

## Discussion

### HIV status disclosure

One objective of this study was to assess the rates of HIV status disclosure and the factors associated with willingness to disclose HIV status. We found a high (79%) rate of HIV disclosure among the 301 participants in this study. We also found that disclosure of HIV took two major forms; direct communication (50%) by the HIV-positive person to family members and disclosure at the health facility (41%) to the family member by health personnel. The high rate of disclosure in this study is consistent with a similar finding that reported a 78.6% prevalence of HIV status disclosure among 510 PLWHA in Cape Coast, Ghana [7]. High rates (95%) were also reported in Cape Town, South Africa among 995 women and in Kenya (83.7%) among 507 PLWHA [8,9]. Our finding is, however, inconsistent with findings in other parts of Ghana where HIV status disclosure was much lower. For example, in a randomized controlled trial in Accra and Kumasi, researchers reported that disclosure was 51.4% in the intervention group and only 16.2% in the controlled group [10]. Again, in the Lower Manya Krobo district of the Eastern region, researchers reported only 33.3% HIV status disclosure to infected children and adolescents [3]. The finding in this study that healthcare professionals disclosed the HIV status of their patients is inconsistent with existing literature and requires further research into the reasons and effects of that route of HIV status disclosure.

Regarding the different kinds of people who gain the trust of PLWHA for HIV status disclosure, we found that 25% of each of the disclosures was to a spouse or sexual partner, parent, and sibling while 14% was to children of the infected person. Spouses or sexual partners were people HIV status was disclosed to in many other findings [7,8,9]. This may be due to close intimacy, shared aspirations, and matrimonial or legal commitments between couples that make spouses/sexual partners confidants for HIV status disclosure.

We found social support to significantly determine HIV status disclosure. This finding is consistent and supported by other findings in the literature. For instance, Obiri-Yeboah et al., found and recommended comprehensive support services as a significant factor for HIV status disclosure [24]. Also, researchers in Ethiopia found among other factors that social support and good spousal relationship were strongly associated with HIV status disclosure [25]. Other factors that are associated with HIV status disclosure are being married and living with a sexual partner [24], regular condom use, and awareness of the sexual partner’s HIV status [26]. However, our finding of ethnicity and religious affiliation as significant predictors of HIV status disclosure is inconsistent with the findings reported here and in the literature and may constitute new knowledge that needs to be researched further. Again, the role of ethnicity and religious affiliation found in this study may be due to communication patterns, nature of relationships, and cultural values placed on ethnicity and religion in the communities we drew our sample from.

It is important to note that 21% of the sample in this study did not disclose their HIV status and fear of family rejection, fear of the spread of HIV status, and fear of community stigmatization and discrimination were reasons for non-disclosure. Other studies have reported similar factors inhibiting HIV status disclosure in Ghana [3,13]. Similar findings in addition to the age of children and the inability to cope with disclosure news have been reported elsewhere [14,15,25]. An analysis of all the reasons provided in the numerous research findings on factors comes to one overarching factor, HIV-related stigma, and all the different forms in which it occurs.

### Experience of HIV-related stigma

In examining the experiences of HIV-related stigma, we found four forms of HIV-related stigma that exist; internalized stigma, family stigma, community stigma, and healthcare/health professional related stigma. We found that healthcare-related (3%) and family-related (4%) HIV stigmas were least experienced in our aggregation of the percentages for all four categories of HIV-related stigma. We found that 109(36%) demonstrated internalized stigma out of which 29(9.6%) reported high internalized stigma. On the other hand, 128(42.5%) reported minimal internalized stigma while 64(21.3%) showed no sign of internalized HIV-related stigma. Bivariate correlation revealed that only gender (r[299] = 0.127, p = 0.02) showed statistically significant correlation with internalized HIV-related stigma while a *Mann-Whitney U-test* revealed that females were significantly more likely to internalize HIV-related stigma compared to males (U = 7426, male = 166.56, female = 145.65, p = 0.05, two-tailed). The results showed that 25% of the participants experienced community-related HIV stigma including being gossiped about (56%) and verbally harassed (31%). Studies on internalized HIV-related stigma are scanty in Ghana and were not also captured in the 2014 GDHS. However, the finding is consistent with the internalized stigma report in the 2014 PLHIV index in Ghana that 41.3% of males and 39.9% of females blamed themselves for their HIV status while a total of 35.3% and 32.7% felt ashamed and guilty [17]. A high prevalence of internalized HIV stigma is also reported in the literature and consistent with our finding in this study [27,28].

However, our finding of community-related HIV stigma against PLWHA is consistent with the finding in the 2014 GDHS. A high prevalence of community stigma against PLWHA was reported in the GDHS as the report revealed that only 8% of women and 14% of men who participated in the survey expressed accepting behaviors towards PLWHA [17]. Researchers also found community-related stigma in four regional capitals in Ghana against HIV-positive men having sex with men (MSM) in the forms of verbal violence (49%), physical violence (13%), and refusal to render services (30%) to PLWHA [29]. Again, Mumin and colleagues reported a high prevalence of internalized, family, community, and institutional HIV-related stigma towards PLWHA in Ghana [30]. Community-related HIV stigma has also been reported outside of Ghana in different countries as researchers reported that half of the population of Nigeria stigmatize and discriminate against PLWHA and a high prevalence of community-related HIV stigma was reported in the Southeastern United States [31,32]. This is consistent with findings in the literature that internalized stigma in particular, and HIV-related stigma, in general, affects HIV serostatus disclosure. For instance, researchers found in Cape Town, South Africa, that internalized HIV-related stigma was associated with less likelihood of HIV status disclosure [8]. HIV-related stigma has also been found to correlate with the nondisclosure of HIV status among African immigrants living in Europe and other parts of the world [33,34,35].

## Limitations

One limitation we encountered was about the confidentiality of personal health information (PHI) whereby some participants feared that their PHI will be disclosed somehow to the public, which could lead to further stigmatization and discrimination. To minimize their fears, we assured all participants that their PHI will never be shared with anybody anywhere and that the data collected was strictly going to be kept at the department under lock and key. We also ensured that the data were collected anonymously using codes instead of personal identifiers, and so no one individual participant could be identified, even by the researchers. Again, during the process of collecting the data, some questions evoked some emotions from participants as some questions appeared sensitive to a few participants. Data collectors occasionally had to allow a few minutes for participants to take breaks to relax before continuing. Nonetheless, we made every effort to ensure the accuracy of the data and ensured the dignity of every participant. We do not believe that these limitations negatively impacted our study and the results presented in this paper.

## Conclusion

The results of this study have revealed a high rate of HIV status disclosure among PLWHA in the three municipalities we covered and social support, ethnicity, and religious affiliation were factors associated with HIV status disclosure. We found that there was significant involvement of healthcare professionals in HIV status disclosure among the participants in this study. Internalized HIV stigma was more prevalent among female PLWHA than males. Our findings suggest that PLWHA are variously affected by stigma and that community-related stigma is a challenge in HIV status disclosure. HIV/AIDS stigma has negative links with testing, treatment, and viral load suppression and is, therefore, a barrier to achieving the 90-90-90 target. Community-level HIV/AIDS stigma can affect health and psychosocial outcomes and is a barrier to disclosure. Strengthening social support systems for PLWHA and developing culturally appropriate community forums to educate community members on the dangers of stigma and discrimination against PLWHA may help in reducing HIV stigma and promote HIV status disclosure. Further research is recommended in the effectiveness and ethics of the involvement of healthcare professionals in HIV status disclosure.

## Additional File

The additional file for this article can be found as follows:

10.5334/aogh.3120.s1 Limited dataset for HIV study. This is the dataset of a broad HIV study from the HIV stigma and status disclosure manuscript was written.

## References

[1] UNAIDS. HIV and AIDS estimate country factsheets: Ghana; 2019. https://www.unaids.org/en/regionscountries/countries/ghana. Accessed June 19, 2020.

[2] Ghana AIDS Commission. People living with HIV (PLHIV) stigma index study Ghana; 8 2014.

[3] Gyamfi E, Okyere P, Appiah-Brempong E, Adjei RO, Mensah KA. Benefits of disclosure of HIV status to infected children and adolescents: Perceptions of caregivers and health care providers. Journal of the Association of Nurses in AIDS Care. 2015; 26(6): 770–780. DOI: 10.1016/j.jana.2015.08.001 26324523

[4] Vreeman RC, Nyandiko WM, Liu H, et al. Measuring adherence to antiretroviral therapy in children and adolescents in western Kenya. Journal of the International AIDS Society. 2014; 17(1): 19227. DOI: 10.7448/IAS.17.1.19227 25427633 PMC4245448

[5] Deribe B, Ebrahim J, Bush L. Outcomes and factors affecting HIV status disclosure to regular sexual partner among women attending antiretroviral treatment clinic. J AIDS Clin Res. 2018; 9(760): 2. DOI: 10.4172/2155-6113.1000760

[6] Hornschuh S, Rice D, Dietrich J. Understanding the HIV status disclosure process, social support structures and antiretroviral therapy adherence among young people in Soweto, South Africa: A qualitative study; 2019.

[7] Dauda RS. HIV/AIDS and economic growth: Evidence from West Africa. The International journal of health planning and management. 2019; 34(1): 324–337. DOI: 10.1002/hpm.2633 30146761

[8] Brittain K, Mellins CA, Remien RH, et al. Patterns and predictors of HIV-status disclosure among pregnant women in South Africa: Dimensions of disclosure and influence of social and economic circumstances. AIDS and Behavior. 2018; 22(12): 3933–3944. DOI: 10.1007/s10461-018-2263-6 30155586 PMC6299267

[9] Colombini M, James C, Ndwiga C, Team I, Mayhew SH. The risks of partner violence following HIV status disclosure, and health service responses: Narratives of women attending reproductive health services in Kenya. Journal of the International AIDS Society. 2016; 19(1): 20766. DOI: 10.7448/IAS.19.1.20766 27037140 PMC4819069

[10] Paintsil E, Kyriakides TC, Antwi S, et al. Clinic-Based Pediatric Disclosure Intervention Trial Improves Pediatric HIV Status Disclosure in Ghana. JAIDS Journal of Acquired Immune Deficiency Syndromes. 2020; 84(1): 122–131. DOI: 10.1097/QAI.0000000000002316 32049772 PMC7138721

[11] Bonnington O, Wamoyi J, Ddaaki W, et al. Changing forms of HIV-related stigma along the HIV care and treatment continuum in sub-Saharan Africa: A temporal analysis. Sexually transmitted infections. 2017; 93(Suppl 3). DOI: 10.1136/sextrans-2016-052975 PMC5739847 28736394

[12] Amo-Adjei J, Darteh EK. Drivers of young people’s attitudes towards HIV/AIDS stigma and discrimination: Evidence from Ghana. African Journal of Reproductive Health. 2013; 17(4): 51–59. 24689316

[13] Poku RA, Owusu AY, Mullen PD, Markham C, McCurdy SA. Considerations for purposeful HIV status disclosure among women living with HIV in Ghana. AIDS care. 2017; 29(5): 541–544. DOI: 10.1080/09540121.2016.1255711 27838933

[14] Kalembo FW, Kendall GE, Ali M, Chimwaza AF. Socio-demographic, clinical, and psychosocial factors associated with primary caregivers’ decisions regarding HIV disclosure to their child aged between 6 and 12 years living with HIV in Malawi. Plos one. 2019; 14(1): e0210781. DOI: 10.1371/journal.pone.0210781 30645639 PMC6333381

[15] Britto C, Mehta K, Thomas R, Shet A. Prevalence and correlates of HIV disclosure among children and adolescents in low-and middle-income countries: A systematic review. Journal of developmental and behavioral pediatrics: JDBP. 2016; 37(6): 496. DOI: 10.1097/DBP.0000000000000303 27262128 PMC5949066

[16] Chan KY, Rungpueng A, Reidpath DD. AIDS and the stigma of sexual promiscuity: Thai nurses’ risk perceptions of occupational exposure to HIV. Culture, health & sexuality. 2009; 11(4): 353–368. DOI: 10.1080/13691050802621161 19263260

[17] Ghana Statistical Service – GSS, Ghana Health Service – GHS, ICF International. Ghana Demographic and Health Survey 2014. Rockville, Maryland, USA: GSS, GHS, and ICF International; 2015.

[18] Beyeza-Kashesya J, Kaharuza F, Ekström AM, Neema S, Kulane A, Mirembe F. To use or not to use a condom: A prospective cohort study comparing contraceptive practices among HIV-infected and HIV-negative youth in Uganda. BMC infectious diseases. 2011; 11(1): 144. DOI: 10.1186/1471-2334-11-144 21605418 PMC3128049

[19] Dessie Y, Gerbaba M, Bedru A, Davey G. Risky sexual practices and related factors among ART attendees in Addis Ababa Public Hospitals, Ethiopia: a cross-sectional study. BMC public health. 2011; 11(1): 422. DOI: 10.1186/1471-2458-11-422 21631935 PMC3138456

[20] Wolfe WR, Weiser S, Bangsberg D, et al. Effects of HIV-related stigma among an early sample of patients receiving antiretroviral therapy in Botswana. AIDS care. 2006; 18(8): 931–933. DOI: 10.1080/09540120500333558 17012082

[21] Godden B. Sample size formulas. Journal of Statistics. 2004; 3(66).

[22] Kothari CR. Research methodology: Methods and techniques. New Delhi: New Age International; 2004.

[23] Frankfort-Nachmias C, Nachmias D. Study guide for research methods in the social sciences.Macmillan; 2007.

[24] Obiri-Yeboah D, Amoako-Sakyi D, Baidoo I, Adu-Oppong A, Rheinländer T. The “fears” of disclosing HIV status to sexual partners: A mixed methods study in a counseling setting in Ghana. AIDS and Behavior. 2016; 20(1): 126–136. DOI: 10.1007/s10461-015-1022-1 25711298

[25] Dessalegn NG, Hailemichael RG, Shewa-Amare A, et al. HIV Disclosure: HIV-positive status disclosure to sexual partners among individuals receiving HIV care in Addis Ababa, Ethiopia. PloS one. 2019; 14(2): e0211967. DOI: 10.1371/journal.pone.0211967 30768642 PMC6415764

[26] Appiah SCY, Adekunle AO, Oladokun A, Dapaah JM, Nicholas KM. Parental Disclosure of Own HIV Status to Children in Two Ghanaian Regions; Examining the Determinants within a Child Vulnerability Context. Health. 2019; 11(10): 1347. DOI: 10.4236/health.2019.1110104

[27] Odiachi A, Erekaha S, Cornelius LJ, et al. HIV status disclosure to male partners among rural Nigerian women along the prevention of mother-to-child transmission of HIV cascade: A mixed methods study. Reproductive health. 2018; 15(1): 36. DOI: 10.1186/s12978-018-0474-y 29499704 PMC5833030

[28] Overstreet NM, Earnshaw VA, Kalichman SC, Quinn DM. Internalized stigma and HIV status disclosure among HIV-positive black men who have sex with men. AIDS care. 2013; 25(4): 466–471. DOI: 10.1080/09540121.2012.720362 23006008 PMC4270468

[29] Dos Santos MM, Kruger P, Mellors SE, Wolvaardt G, Van Der Ryst E. An exploratory survey measuring stigma and discrimination experienced by people living with HIV/AIDS in South Africa: The People Living with HIV Stigma Index. BMC public health. 2014; 14(1): 80. DOI: 10.1186/1471-2458-14-80 24461042 PMC3909177

[30] Mumin AA, Gyasi RM, Segbefia AY, Forkuor D, Ganle JK. Internalised and social experiences of HIV-induced stigma and discrimination in urban Ghana. Global Social Welfare. 2018; 5(2): 83–93. DOI: 10.1007/s40609-018-0111-2

[31] Dahlui M, Azahar N, Bulgiba A, et al. HIV/AIDS related stigma and discrimination against PLWHA in Nigerian population. PloS one. 2015; 10(12): e0143749. DOI: 10.1371/journal.pone.0143749 26658767 PMC4675522

[32] Baugher AR, Beer L, Fagan JL, et al. Prevalence of internalized HIV-related stigma among HIV-infected adults in care, United States, 2011–2013. AIDS and Behavior. 2017; 21(9): 2600–2608. DOI: 10.1007/s10461-017-1712-y 28213821 PMC5555833

[33] Whembolua G-L, Conserve DF, Thomas K, Handler L. A systematic review of HIV serostatus disclosure among African immigrants in Europe. Journal of immigrant and minority health. 2017; 19(4): 947–958. DOI: 10.1007/s10903-016-0456-5 27388442

[34] Stutterheim SE, Brands R, Baas I, Lechner L, Kok G, Bos AE. HIV status disclosure in the workplace: Positive and stigmatizing experiences of health care workers living with HIV. Journal of the Association of Nurses in AIDS Care. 2017; 28(6): 923–937. DOI: 10.1016/j.jana.2017.06.014 28751112

[35] Olley B, Ogunde M, Oso P, Ishola A. HIV-related stigma and self-disclosure: The mediating and moderating role of anticipated discrimination among people living with HIV/AIDS in Akure Nigeria. AIDS care. 2016; 28(6): 726–730. DOI: 10.1080/09540121.2016.1140894 26882476

